# Stochastic Models of Emerging Infectious Disease Transmission on Adaptive Random Networks

**DOI:** 10.1155/2017/2403851

**Published:** 2017-09-17

**Authors:** Navavat Pipatsart, Wannapong Triampo, Charin Modchang

**Affiliations:** ^1^Department of Physics, Faculty of Science, Mahidol University, Bangkok 10400, Thailand; ^2^Centre of Excellence in Mathematics, CHE, Bangkok 10400, Thailand; ^3^Thailand Center of Excellence in Physics, CHE, 328 Si Ayutthaya Road, Bangkok 10400, Thailand

## Abstract

We presented adaptive random network models to describe human behavioral change during epidemics and performed stochastic simulations of SIR (susceptible-infectious-recovered) epidemic models on adaptive random networks. The interplay between infectious disease dynamics and network adaptation dynamics was investigated in regard to the disease transmission and the cumulative number of infection cases. We found that the cumulative case was reduced and associated with an increasing network adaptation probability but was increased with an increasing disease transmission probability. It was found that the topological changes of the adaptive random networks were able to reduce the cumulative number of infections and also to delay the epidemic peak. Our results also suggest the existence of a critical value for the ratio of disease transmission and adaptation probabilities below which the epidemic cannot occur.

## 1. Introduction

Within few decades, numerous studies on infectious disease transmission using the network theory have been carried out. Networks provide a mathematical platform for the interpretation of the interaction between individuals or populations and are especially useful when each individual is assumed to be in direct contact only with a small proportion of the population [1–5]. Network models tend to be very powerful tools that provide understanding of the disease transmission in human populations and allow the assumptions of either social or sexual contacts [6]. However, the vast majority of infectious disease transmission models on networks employed static networks [7]. The static network structure does not change over time and such models ignore the effect of individual's behavioral change due to the infection. On the other hand, there are studies that implemented rules on dynamical network structures that opened the possibility of network adaptation. These rules help to generate complex network models and are also expected to reflect some real-world networks [8–13].

Recent studies brought forth characteristic rules of networks that adapted the network structure more dynamically by responding to the infection status of individuals [14–16]. These dynamic networks took into account the fact that individuals tend to respond to the emerging infectious disease transmission by avoiding contacts with infected individuals. Such rewiring of local contacts can have a strong impact on the dynamics of an infectious disease transmission as shown in a study of a complicated mutual interplay between network adaptation dynamics and the dynamics of individual states. There are two popular closely related adaptive rules of the network: the first rule allows susceptible individuals to temporarily disconnect their contacts with infectious individuals [17] and the other allows susceptible individuals to avoid contact with the infectious individuals by rewiring their network connections [18]. This has revealed new perspectives onto effects on concurrent partnerships [19, 20] and on structure changing patterns [21]. A definition of dynamic networks was given stating that such networks are regulated by a feedback loop between the dynamics of node's state and interaction in a network and the coevolution or adaptation of the networks [22].

Most studies on infectious disease transmission using adaptive networks are deterministic. Admittedly, the implementation of such models is easier, but they are insufficient to explain some fluctuating dynamics in real-world systems [23]. Deterministic models provide exactly the same results given the same initial conditions. However, we would not expect to observe exactly the same people becoming infected at exactly the same time. In contrast, infectious disease modeling using the adaptive networks takes into account the fluctuations or noise by considering interactions between individuals. Clearly, there is an important element of chance and stochastic models are concerned with approximating this random or probabilistic element. In general, chance will play the most important role whenever the number of infectious individuals is relatively small, which can happen when the population size is small. It is especially important that stochasticity is taken into account and incorporated into the network model.

However, there is no comparative study about effect of these two adaptive rules to the infectious disease transmission in population yet. The main goals of this work is not only to model networks representing individuals in a population that tend to respond to the emergence of an infectious disease and incorporate two different specific patterns of behavioral change regarding the interaction between individuals in a population but also to investigate the interplay between infectious disease dynamics and network adaptation dynamics by using stochastic simulations in order to gain insight into infectious disease transmission in real-world system.

## 2. Network Models

### 2.1. Construction of Static Random Network

In our model, we represented an individual human by a node and a potential disease-causing interaction between two individuals by a link. The *G*(*n*, *p*) random network was constructed from a finite set of *n* nodes with *n*(*n* − 1)/2 possible pairs. Each pair of nodes was then randomly chosen to be connected with a probability *p*, where the number of nodes *n* and independent probability *p* are fixed. We defined the initial structure of random network at each simulation run by setting the initial structure as a *G*(*n*, *p*) random network. The number of individuals in this system was fixed at *n* = 1,000 nodes. We expected a low frequency of the disease-causing contact between two individuals henceforth, comprising a small proportion of the population and represented by a low probability *p* = 0.01.

### 2.2. Infectious Disease Transmission on Networks

According to the* SIR* epidemic model mechanism, an individual human was represented as a node amongst a finite and fixed number of individuals in a population and the potential disease-causing contact between two individuals as a link. Two nodes were assigned as neighbors, if connected by a link. A node neither could be linked to itself nor shares more than one link with another node. At any time, each node has only one specific state, namely, the susceptible state (*S*), the infectious state (*I*), or the recovered state (*R*). This model is appropriate for a disease that spreads through human populations by direct contact between infectious individuals and susceptible individuals, such as influenza. We also assumed that recovered individual confers lifelong immunity. In this paper we will consider only diseases of this type. Diseases that are endemic because they propagate on time scales comparable to or slower than the rate of turnover of the population or because they confer only temporary immunity are not well represented by this model.

For the *S*→*I* transition, an infectious disease can be transmitted, if a given infectious node shares links with other susceptible nodes. We denoted this link as *SI*-link. Those susceptible neighbors were then infected with probability *ϕ* per *SI*-link and per time-step. In addition, the more the contacts a susceptible node has with infectious nodes, the higher the chance to become infected simply is because a pathogen can be transmitted from many infected individuals. Hence, we calculated the infection probability *λ* of each node individually as follows:

(1) $$\begin{array}{c} \lambda =1-{\left(\;1-\phi \;\right)}^{k_{SI}}, \end{array}$$

where *k*_*SI*_ is the number of *SI*-links that is connected to that node. The *I*→*R* transition implies that infected nodes can independently self-recover with a recovery probability *η*. In contrast to the *S*→*I* transition, the *I*→*R* transition is not influenced by *SI*-links. The initial conditions were set as follow: (1) The entire nodes in the network are in susceptible state. (2) A single node is randomly chosen to become the first infectious node.

**Figure 1:**
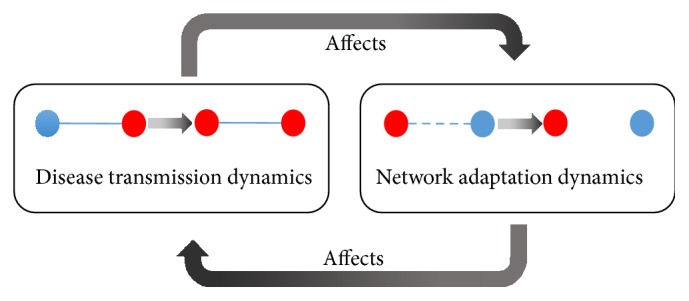
Schematic diagram of the feedback loop between the infectious disease dynamics and network adaptation dynamics of an adaptive network. Blue and red nodes represent susceptible and infectious nodes, respectively. Solid and dot lines represent network links and links that will be cut due to network adaptation dynamics, respectively.

When an infectious disease spreads on a social network, humans tend to respond to the emergence of an epidemic by avoiding contacts with infected individuals. Such rewiring of the local connections can have a strong effect on the dynamics of the disease, which in turn influences the rewiring process. Thus, a complicated mutual interaction between network adaptation dynamics and the dynamics of disease transmission emerges (Figure 1). In our network, such nodes with reconnecting or rewiring ability were called adaptive nodes. It shall be noted that only susceptible nodes are capable of such adaptation once being at risk of infection. Hence, the network adaptation concerned the ability of susceptible nodes to adapt as to avoid contact with infectious nodes. In addition, these behavioral changes can lead to the isolation of infected nodes and then again affects the behavioral changes of susceptible nodes. Consequently, there is interplay between the changing state of nodes and the changing interaction of nodes at any time of infectious disease transmission on the network. This provides a feedback loop between the infectious disease dynamics and network adaptation dynamics of the network as illustrated in Figure 1. These networks including feedback loops are defined as coevolution networks or adaptive networks [24].

Thus, this feedback loop is dependent on the existence of a pathogen, which can trigger node state changes and altered interaction of nodes. This feedback loop then determines the outcome of the infectious disease transmission on the network, which is either invasion or disappearance of the pathogen. At any given time of the ongoing infectious disease transmission on an adaptive network, both changing states of nodes and their interaction have potential to occur. The dynamics also lead to a new perspective that cannot be observed using a static network model. There are two types of adaptive networks in this work, which are described in the following.

**Figure 2:**
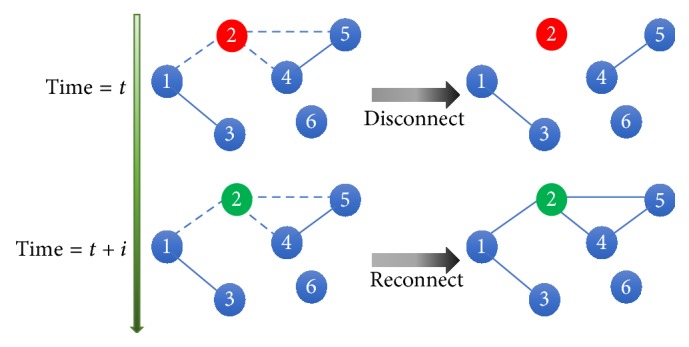
Schematic illustration of the adaptation process of type I adaptive network. At time *t*, the susceptible nodes 1, 4, and 5 can disconnect their *SI*-links, namely, (1,2), (2,4), and (2,5) links, with the probability *ω*_I_. At time *t* + *i*, when the node 2 recovers from the disease, the disconnected links will be reconnected. Blue, red, and green nodes represent susceptible, infectious, and recovered nodes, respectively. Dot lines represent links that are disconnected and then reconnected.

*Type I Adaptive Random Network.* In this model, the adaptive and susceptible nodes have the feature to protect themselves by temporarily disconnecting each of their *SI*-link between infectious neighboring nodes with probability *ω*_I_ at any time-step. The destroyed links are then reconnected when the neighboring nodes transform into recovered nodes, as shown in Figure 2.

In this way, every susceptible node has a constant and equal chance to disconnect each of their *SI*-links and then also reconnects the formerly destroyed links at every time-step. Therefore, the number of links, *m* in this network, is changed in accordance with the time-steps. Type I adaptation in an adaptive random network represents a real-world situation given the assumption of effective quarantine of infected people during epidemics and the later release after their recovery from infection.

*Type II Adaptive Random Network.* The network adaptation dynamics of this model allows adapting susceptible nodes to protect themselves by rewiring their links. The adaptive and susceptible nodes disconnect their *SI*-links with the infectious nodes with probability *ω*_II_ for every *SI*-link at each time-step. This rule of rewiring is basically similar to the type I adaptive network; however, instead of reconnecting with the former neighbors, other susceptible or recovered state nodes will be randomly chosen to establish new links immediately after successful disconnection of the previous *SI*-links. The chosen nodes are prohibited to link with themselves and their current neighboring nodes. We define this behavioral change as rewiring of the links and denote *ω*_II_ as the rewiring probability.

**Figure 3:**
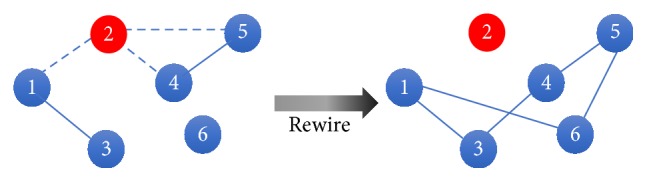
Schematic illustration of the adaptation process of type II adaptive network. In this figure, nodes 1, 4, and 5, can cut their (1,2), (2,4), and (2,5) links with the probability *ω*_II_. The node that decided to cut its *SI*-link will immediately choose a new noninfectious node to connect with. Blue, red, and green nodes represent susceptible, infectious, and recovered nodes, respectively. Dot lines represent *SI*-links.

However, every susceptible node in this model does not memorize their prior destroyed links and their abandoned neighbors. Consequently, adaptive and susceptible nodes can either reconnect to the abandoned neighboring nodes or any other nodes at later time-step as shown in Figure 3.

At any time-step, the adaptive and susceptible nodes responded to the disease transmission by rewiring their links, but the number of links *m* remained constant. On the other hands, the behavioral change of individual nodes in regard to the avoidance of contact with infected individual nodes is determined by destruction probability *ω*_I_ and rewiring probability *ω*_II_ for the type I adaptive random network and the type II adaptive random network, respectively. The value range of both the epidemic and the network adaptation probabilities are shown in Table 1. All simulations were performed using the standard kinetic Monte Carlo algorithm and were simulated using MATLAB software.

## 3. Results

### 3.1. Infectious Disease Transmission on Type I Adaptive Random Networks

**Figure 4:**
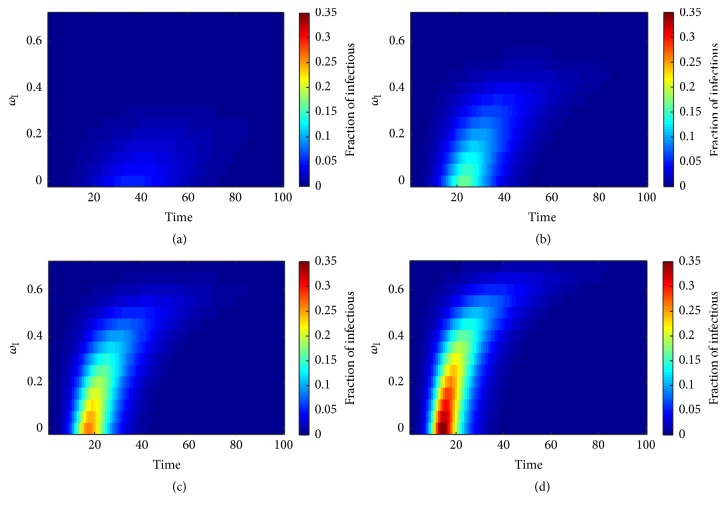
Profile of the infectious disease's prevalence plotted against time and destruction probability *ω*_I_ on the type I adaptive random network. The ratio *χ* ≡ *ϕ*/*η* was varied as (a) *χ* = 2.0, (b) *χ* = 3.0, (c) *χ* = 4.0, and (d) *χ* = 5.0. Grids correspond to the average of over 5,000 simulations.

This section presents the results of the investigations on the behavior of the infectious disease transmission on type I adaptive random network upon variation of the network adaptation probability or more precisely the destruction probability *ω*_I_. We assigned other parameters according to Table 1. Then, we have allowed the chosen infected node to transmit the pathogen across its *SI*-links. We investigated the disease transmission over a period of 100 time-steps and varied *ω*_I_ from 0 to 0.7, while keeping ratio *χ* ≡ *ϕ*/*η* constant (Figure 4). Colored labeling of the fraction of infectious nodes was applied to facilitate the illustration.  Figure 4(a) shows the results for *χ* = 2.0. We found that the pathogen failed to invade the population. A minor occurrence of an infectious fraction can be observed, if *ω*_I_ < 0.3. The infectious fraction tended to be decreased in case of the static network model (*ω*_I_ = 0).

The effect of the network adaptation probability is more obvious, if the pathogen has a higher potential to be transmitted as show in Figure 4(b). We can see that the appearance of the infectious fraction tended to be delayed with increasing *ω*_I_ to the point of its disappearance for *ω*_I_ exceeding 0.50. Independent of the delay, the infectious fraction tended to last until stationary state of the network at similar time-steps and measured an average period of appearance of 47 time-steps. The appearance of the infectious fraction in Figure 4(c) followed previous patterns observing the delay in association with an increasing value of *ω*_I_ and a fixed *χ* = 4.0. The infectious fractions occurred at time-steps 6, 7, 7, 8, 10, 13, and 20 for *ω*_I_ = 0.1, 0.2, 0.3, 0.4, 0.5, and 0.6, respectively, and then decayed until stationary state. The fractions of infectious nodes tended to be reduced in case of the static network model. The average period of the appearance was 41 time-steps ranging from 33 to 52 time-steps for values of *ω*_I_ ranging between 0 and 0.55. The period of appearance measured 45 time-steps in case of *ω*_I_ = 0.60. These results led us to realize that the successful disease invasion did depend not only on the network adaptation probability, which is a parameter of individual behavioral change, but also on the disease transmission ratio *χ*, which is a parameter of the infectious disease, too. More precisely, the infectious disease can invade the population for specific values of both *ω*_I_ and *χ*.

Figure 4(d) shows the time evolution of the fraction of infected nodes and the appearance of invasion of the disease for the entire range of *ω*_I_ while *χ* was fixed at 5.0. Therefore, this model tends to speed up the disease transmission but reaches the stationary state at similar time-points when compared to previous results. On the other hand, the periods of appearance tend to be increased with an increasing value of *ω*_I_, except for *ω*_I_ = 0.7. We noticed that infectious fraction began to appear and the commencement of the stationary state was slightly delayed with increasing *ω*_I_. In the same way, the infectious fractions tended to decline in the static network model with increasing *ω*_I_ until their disappearance using an appropriate value of *ω*_I_. Also, the periods of appearance of the infectious fractions tended to increase with increasing *ω*_I_ and could be reduced by choosing an appropriate value of *ω*_I_. It must be noted that the behavioral change of individuals as modeled in the type I adaptive random network is reflected by network adaptation dynamics and may have caused the reduction in numbers of infectious people in a population. Also, such dynamics can protect susceptible individuals from infection by avoiding contact with infectious people. The higher the chance of avoidance, the lower the number of infectious individuals in that population. In this way, the destruction probability *ω*_I_ can be interpreted as the chance of susceptible individual to detect infected neighbors in a local population. Thus, *ω*_I_ is a parameter to determine behavior of individual.

The results show that the infectious disease can invade the population, if the pathogen transmission is probable despite a 70-percent chance of any susceptible individual in the population to avoid contact with infected individuals. Furthermore, the behavioral change of susceptible individuals as represented by the network adaptation dynamics in type I adaptive random networks has potential to reduce the number of infected people prospectively but also can lead to a delay in the epidemic peak time. In this way, the individual behavior may lead to a prolonged disease spread in that population.

### 3.2. Infectious Disease Transmission on Type II Adaptive Random Networks

**Figure 5:**
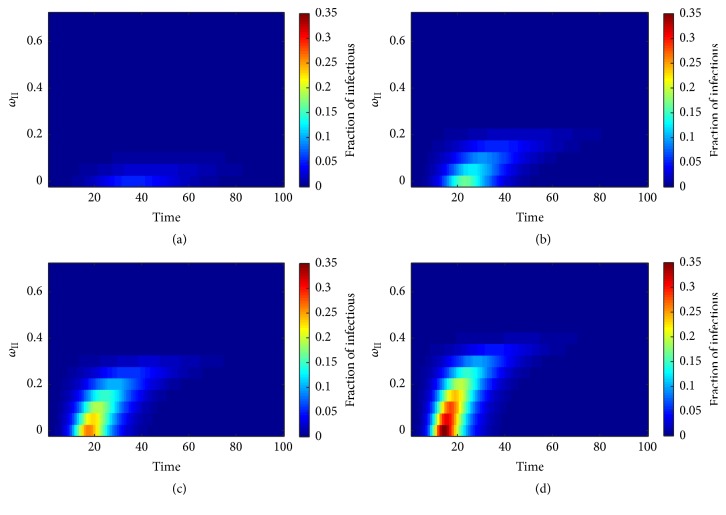
Profile of the infectious disease's prevalence plotted against time and destruction probability *ω*_II_ on the type II adaptive random network. The probability ratio *χ* was varied as (a) *χ* = 2.0, (b) *χ* = 3.0, (c) *χ* = 4.0, and (d) *χ* = 5.0. Grids correspond to the average of over 5,000 simulations.

Thereafter, we studied the infectious disease transmission in another adaptive network model, namely, the type II adaptive random network, by using the equivalent conditions as in the previous section to investigate the effect of network adaptation dynamics on the infectious disease transmission.  Figure 5(a) shows the time evolution of fractions of infectious nodes with varying *ω*_II_ and fixed *χ* = 2.0. The appearance of the infectious fractions is clearly visible (colored pixels against blue background) in case of the static network model and type II adaptive random network for *ω*_II_ = 0.05. This corresponds to the results in a previous section. This stands in contrast to the behavior of the type I network, which produced disease invasion only for a network adaptation probability less than 0.3.  Figure 5(b) shows the time evolution of infectious fractions for *χ* = 3.0. Infectious fractions appeared for *ω*_II_ ≤ 0.2 and tended to be increased with proceeding time-steps arriving at an epidemic peak and then decayed to zero at stationary state. In case of the static network model, the infectious fraction tended to decrease dramatically. In the case of adaptive networks, the infectious fraction decreased with an increasing *ω*_II_ until its disappearance for *ω*_II_ > 0.2. The time-steps of occurrence of the infectious fractions tended to be delayed with an increasing *ω*_II_ and infectious fractions remained in the network for an average period of 46 time-steps. In this case, the period of appearance was similar to the simulation results of the type I adaptive network measuring 40–45 time-steps. Nevertheless, the fraction of infected nodes was always lower when compared to the type I adaptive network under equivalent conditions.

Moreover, Figure 5(c) shows the simulation result for *χ* = 4.0. The results followed previous patterns observing delayed occurrence of the infectious fraction, but its period tended to be increased with an increasing *ω*_II_. However, the stationary state of infectious fractions tended to be delayed for higher values of *ω*_II_. The time evolution of the infectious fraction became obvious for *ω*_II_ ≤ 0.30. We noticed a shift in the limitation of occurrence of the infectious fractions when the disease transmission ratio *χ* was increased by 0.5 points. This might be due to the interplay between the network adaptation probability and the disease transmission ratio. Moreover, the average period of appearance of infectious fractions tended to be decreased by about 6 time-steps when compare to the previous results.

In addition, Figure 5(d) shows the simulation results for *χ* = 5.0. Infectious fractions appeared, if the rewiring probability *ω*_II_ ranged between 0 and 0.4. Fractions and also the network stationary state tended to be delayed with the increase in *ω*_II_. The period of appearance of infectious fractions tended to increase with the increase in *ω*_II_ except in case of *ω*_II_ = 0.40, which measured only 22 time-steps. This might be due to the observation of a large fluctuation. Consequently, the infectious fractions as simulated for the type II adaptive random network were less obvious than in case of the type I network under same parameter conditions. Particularly, when the network adaptation probability was above 0.4, the infectious disease failed to invade the population in case of the type II network for every value of *χ*, whereas succeeded in all ranges of *χ*, in case of type I network simulations. The infectious fractions in the type II case tended to occur later but remained longer when compared to the type I network for equivalent parameters.

Furthermore, we observed that the infectious disease transmission in the adaptive networks was less frequent when compared to the static network simulations in regard to an increasing value of the network adaptation probability. For the infectious disease transmission in type I adaptive random networks, susceptible nodes continued to forsake their infectious neighboring nodes. To follow the thought, we can assume an increasing number of isolated infected nodes. Relations have reformed when the infected nodes have recovered. Similar to the infectious disease transmission on type II adaptive random networks, susceptible nodes continued to change connections by seeking contact with noninfectious nodes upon linkage with infectious neighboring nodes. This contributes to a form of community, which excludes any infected nodes. This particular behavior may well reduce the numbers of disease transmissions and yet also delays the period of disease transmissions. Moreover, in contrast to type I, type II adaptive networks comprise a more sensitive topology in regard to the network adaptation probability. It is meant that it can reduce the number of infections but delays the disease transmission and remission. We explain this with the remaining and more frequent paths of pathogen transmission in the type I adaptive network than can be found in type II networks, despite the destructions of numerous infection paths.

### 3.3. Effects of Network Adaptation Probability to the Infectious Disease Transmission on Adaptive Random Networks

**Figure 6:**
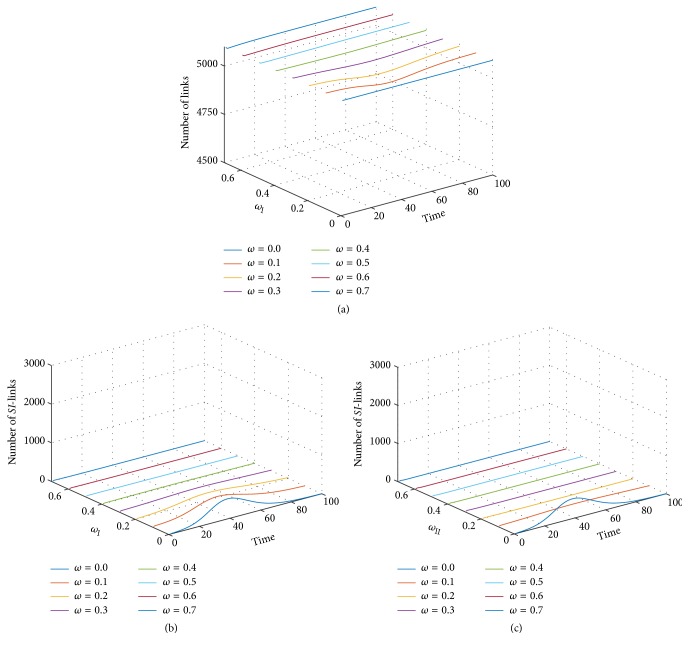
Profile of time evolution of the number of links in type I adaptive random networks (a), the number of *SI*-links in type I adaptive random networks (b), and the number of *SI*-links in type II adaptive random networks (c) (*χ* = 2.0).

In order to understand the effects of network adaptation probabilities on the infectious disease transmission, we plotted the number of links and the number of *SI*-links of both types of adaptive networks against time as shown in Figure 6; the figure shows the simulation result based on varying values of the disease transmission ratio *χ* ranging from 2.0 to 5.0. As described in previous sections, we have learned that the number of links in the type II adaptive random network is constant due to the topology of the network adaptation dynamics. The number of initial links counted 5,094 links. Figure 6 shows the time evolution of links of the type I and the type II adaptive random networks where *χ* was set to 2.0. The number of links in the type I network was reduced for *ω*_I_ ≤ 0.20. At time-steps 43, 49, and 53, 34, 38, and 23 links were disconnected for *ω*_I_ = 0.1, 0.2, and 0.3, respectively, as show in Figure 6(a). In addition, there are 338, 189, and 75 *SI*-links in the type I network at time-steps 43, 48, and 53 for *ω*_I_ = 0.1, 0.2, and 0.3, respectively, as shown in Figure 6(b). We noticed that these time-steps are correlated with each other and coincided with epidemic peaking calling them epidemic peak time. Previous results showed that the infectious disease failed to invade for *ω*_I_ = 0.30 which might be correlated with the number of disconnected *SI*-links. Furthermore, the time evolution of *SI*-links in the type II adaptive random network is shown in Figure 6(c). We observed that almost all of the *SI*-links were rewired before the pathogen was transmitted. This may have had effect on the invasion of infectious disease in the type II network and may have contributed to the failure of invasion.

**Figure 7:**
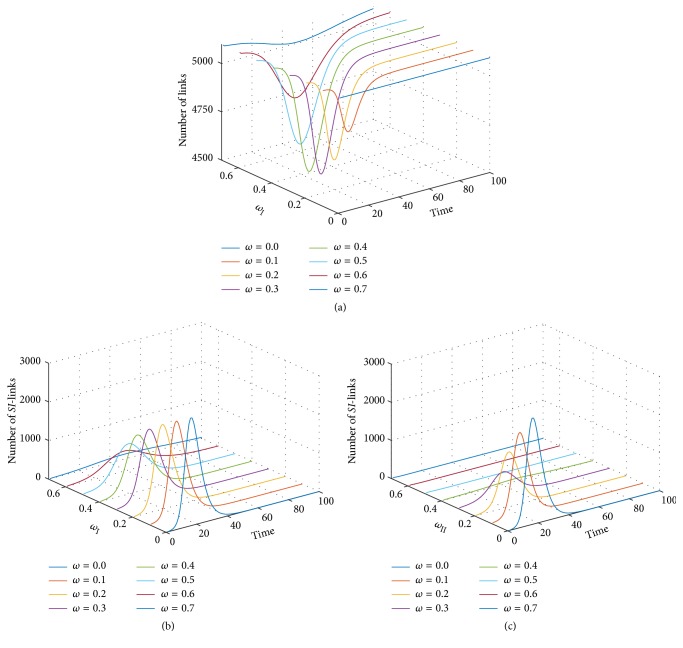
Profile of time evolution of the number of links in type I adaptive random networks (a), the number of *SI*-links in type I adaptive random networks (b), and the number of *SI*-links in type II adaptive random networks (c) (*χ* = 5.0).

Further support of the obtained pattern of results provides the time evolutions of the number of links in the type I model and the number of *SI*-links in the type I and type II network models for *χ* = 5.0 as shown in Figure 7. The number of disconnected links tended to be slightly increased when compared to the previous results. In addition, the number of *SI*-links of both the type I and II networks was also comparatively higher.

According to our results, the change of interaction based on the network adaptation dynamics had strong effect on the reduction of the number of *SI*-links. Choosing an appropriate value for the network adaptation probability was crucial for the failure of the infectious disease invasion due to the destruction or rewiring of most *SI*-links before the pathogen could be transmitted. The number of disconnected links depended on the number of *SI*-links in the network. It was found that the higher the network adaptation probability, the higher the fraction of disconnected *SI*-links. Consequently, this provided a way to think about the failure phenomenon of infectious disease invasion even if it has a high capacity of transmission.

### 3.4. Interplay between Network Adaptation Probability and Disease Transmission Ratio and Its Effect on the Infectious Disease Transmission on Adaptive Random Networks

**Figure 8:**
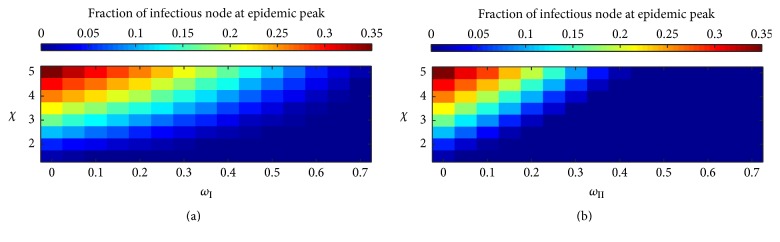
Profile of the infectious fraction at the epidemic peak time as function of the network adaptation probabilities *ω*_I_ and *ω*_II_ and the disease transmission ratio *χ* of type I adaptive random network (a) and type II adaptive random network (b).

In this work, we also studied the interplay between the varied values of the network adaptation probability and the disease transmission ratio in the 2 types of adaptive networks. We observed that in the previous sections an increase of the infectious fraction reaches its peak at the epidemic peak time and then decayed to zero at the stationary state after an appropriate period of time-steps. More precisely, keeping the disease transmission ratio *χ* constant and increasing the network adaptation probabilities *ω*_I_ and *ω*_II_ affected the epidemic peak time and the infectious fraction at epidemic peak time by delaying the time of occurrence and reducing the fraction nodes. On the other hand, it hastened the time of epidemic peaking, if the network adaptation probabilities *ω*_I_ and *ω*_II_ were fixed and the disease transmission ratio *χ* was increased. We observed competition between the values of *ω*_I_ and *ω*_II_ and *χ* affecting the incidence of epidemic peaking of the infectious fraction by increasing *χ* but decreasing *ω*_I_ and *ω*_II_. We plotted the infectious fraction against the disease transmission ratio and the network adaptation probability to obtain more understanding of the interplay between these probabilities. From the results in previous sections, we observe that the epidemic peak time was dramatically delayed in case of the static network simulations.  Figure 8 shows the infectious fraction at epidemic peak time-steps in cases of the type I and II network models. We observe a tentatively decrease of the infectious fraction at epidemic peak time in the static type I network but a dramatically decrease in case of simulations of the type II network with an increasing value of the network adaptation probability. The pattern of the colored grids tended to behave in a linear fashion with an increase in *ω*_I_ and *ω*_II_ and *χ* in both cases of type I and II network models. In this way, there is a connection line between dark blue grids and nondark blue grids. It refers to a critical value below which the infectious disease is considered to fail its invasion. This led us to the description of the threshold phenomenon.

Consequently, the epidemic peak time of the type I adaptive network occurs earlier and its infectious fraction at epidemic peak time is larger than similar simulations using the type II adaptive network.

**Figure 9:**
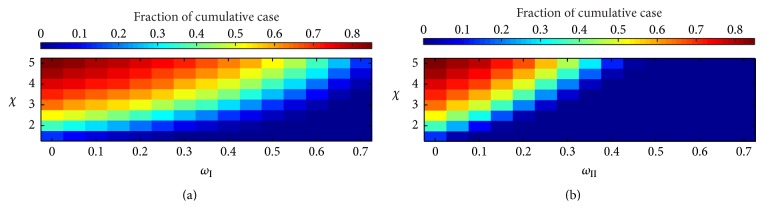
Profile of the cumulative cases as function of the network adaptation probabilities *ω*_I_ and *ω*_II_ and the disease transmission ratio *χ* of type I adaptive random network (a) and type II adaptive random network (b).

The results tell that the interplay between the network adaptation probability and the disease transmission ratio affects the epidemic peak time, the infectious fraction at the epidemic peak time, and also the cumulative number of infection cases at stationary state *R*_*∞*_ as shown in Figure 9. The cumulative number of infection cases at stationary state, *R*_*∞*_, for calculations with the static random network ranged from 0.14 to 0.85 and the disease transmission ratio *χ* ranged between 1.5 and 5.0. In this set of results, a basically similar pattern was observed between both the type I and II network models. More precisely, the number dramatically decreased with an increasing network adaptation probability but dramatically increased with an increasing disease transmission ratio. We learned that the infectious disease is expected to fail invasiveness, if there are less than 10 cumulative cases of infection at stationary state. The threshold phenomenon then occurred for appropriate probabilities in both cases of type I and II network models. Hence, the threshold condition for the type I network was a destruction probability *ω*_I_ in range of 0.3 to 0.55 and a disease transmission ratio *χ* less than 3 with a 3.95 slope. Interestingly, the threshold condition for the type II networks was fulfilled for the entire range of the ratio between the rewiring probability *ω*_II_ and the disease transmission ratio *χ* measuring a slope of 8.65. In any cases of the emerging infectious disease transmission, the number of cumulative case fractions in the type I adaptive random network is higher than in the type II adaptive random network.

**Figure 10:**
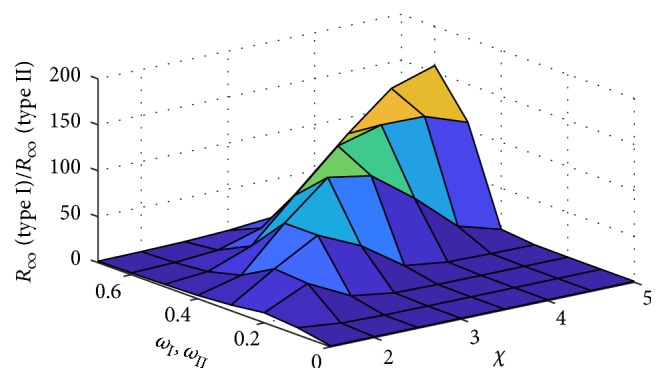
Profile of relative cumulative cases of type I and type II adaptive network against network adaptation probabilities *ω*_I_ and *ω*_II_ and epidemic characteristic ratio *χ*. *R*_*∞*_ is the number of recovered individuals at the equilibrium.

Finally, we investigated the relative cumulative cases of type I and type II adaptive random networks at equivalent conditions as shown in Figure 10. This exhibits a difference of interplay between network adaptation probability and the disease transmission ratio. We plotted the relative cumulative cases against the network adaptation probabilities *ω*_I_ and *ω*_II_ and the disease transmission ratio *χ*. The curve tended to increase with increasing *ω*_I_ and *ω*_II_ along the *χ*-axis but obviously vanished for *ω*_I_ and *ω*_II_ exceeding the value of 0.6. In addition, there was a high ratio number between cumulative cases of type I and type II adaptive network for *ω*_I_ and *ω*_II_ ranging between 0.4 and 0.6, because it was below the threshold condition of type II adaptive network in these cases.

Moreover, with an appropriate value setting of *χ* and *ω*_I_ and *ω*_II_ the infectious disease failed to invade the population. This led us to the threshold conditions for these models in which other parameters and structures of the initial network condition were fixed. The threshold parameter is supposed to intersect between dark blue and semidark blue grids and to be a function of both *χ* and *ω*_I_ and *ω*_II_. In addition, the topology of network adaptation dynamics in the type II adaptive random network reduced more effectively the disease transmissions than the topology of network adaptation dynamics in the type I adaptive network. In contrast to the type II adaptive random network, the number of links in the type I adaptive random network changed over time, due to the destroyed links of the susceptible nodes. This effect reduced the average degree of nodes in such networks. The epidemic peak time was shown to exhibit a maximum number of destroyed *SI*-links and was always equal to the epidemic peak time of infectious fraction, because there was a maximum number of *SI*-links at that time, while the susceptible nodes attempted to disconnect their *SI*-links until reaching the stationary state. Consequently, we may argue that if the emerging infectious disease has a high transmissibility, the individuals in the population must increase their chance to avoid contact with infected individuals in order to protect the population from the invasion.

## 4. Conclusion

In this research, the modeling of adaptive random networks was studied. We concluded that the topology of network adaptation dynamics may have strong effect on reducing the infectious disease transmission on respective network models. The number of cumulative cases of infection at stationary state was affected by both epidemic parameters and adaptive parameters. The networks with adaptation dynamics such as the type II adaptive random network are more effective in reducing the final epidemic spread when compared with the type I adaptive random network. Moreover, the results suggested the occurrence of a threshold phenomenon independence of choices of appropriate values of respective network parameters.

## Figures and Tables

**Table 1:** The value range of the parameters that were used for the simulation.

Time, *t*	1–100
Disease transmission ratio, *χ*	1.5–5.0
Recovery probability, *η*	0.2
Destruction probability, *ω*_I_	0.0–0.7
Rewiring probability, *ω*_II_	0.0–0.7
